# Multi-objective optimization as a tool to identify possibilities for future agricultural landscapes

**DOI:** 10.1016/j.scitotenv.2019.06.070

**Published:** 2019-10-15

**Authors:** Lindsay C. Todman, Kevin Coleman, Alice E. Milne, Juliana D.B. Gil, Pytrik Reidsma, Marie-Hélène Schwoob, Sébastien Treyer, Andrew P. Whitmore

**Affiliations:** a Rothamsted Research, Harpenden, Hertfordshire AL5 2JQ, UK; b Plant Production Systems group, Wageningen University, the Netherlands; c Institut du Développement Durable et des Relations Internationales (IDDRI), 41 Rue du Four, 75006 Paris, France

## Abstract

Agricultural landscapes provide many functions simultaneously including food production, regulation of water and regulation of greenhouse gases. Thus, it is challenging to make land management decisions, particularly transformative changes, that improve on one function without unintended consequences for other functions. To make informed decisions the trade-offs between different landscape functions must be considered. Here, we use a multi-objective optimization algorithm with a model of crop production that also simulates environmental effects such as nitrous oxide emissions to identify trade-off frontiers and associated possibilities for agricultural management. Trade-offs are identified in three soil types, using wheat production in the UK as an example, then the trade-off for combined management of the three soils is considered. The optimization algorithm identifies trade-offs between different objectives and allows them to be visualised. For example, we observed a highly non-linear trade-off between wheat yield and nitrous oxide emissions, illustrating where small changes might have a large impact. We used a cluster analysis to identify distinct management strategies with similar management actions and use these clusters to link the trade-off curves to possibilities for management. There were more possible strategies for achieving desirable environmental outcomes and remaining profitable when the management of different soil types was considered together. Interestingly, it was on the soil capable of the highest potential profit that lower profit strategies were identified as useful for combined management. Meanwhile, to maintain average profitability across the soils, it was necessary to maximise the profit from the soil with the lowest potential profit. These results are somewhat counterintuitive and so the range of strategies supplied by the model could be used to stimulate discussion amongst stakeholders. In particular, as some key objectives can be met in different ways, stakeholders could discuss the impact of these management strategies on other objectives not quantified by the model.

## Highlights

• Trade-offs between different objectives in agricultural landscapes are complex.
• Cluster analysis helped visualise effects of management on trade-offs.
• Minimum N_2_O emissions scaled linearly with yield until ~85–90% of maximum yield.
• A more fertile soil could be managed more flexibly and remain profitable.
• Achieving profitability on the *least* fertile soil was key for overall profitability.

## Graphical abstract

**Figure:**
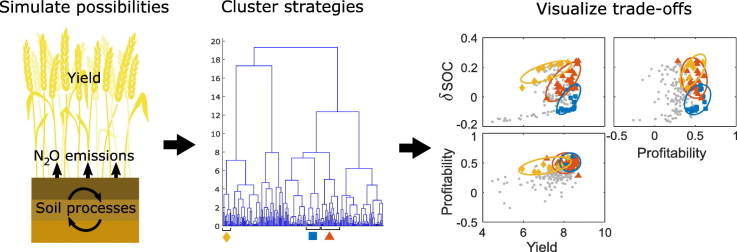

## 1 Introduction

The United Nations Sustainable Development Goals (SDGs) set out an ambitious suite of targets to stimulate effort to improve sustainability globally. Core to the SDGs is that these targets should not be considered in isolation, but that the interlinkages between the goals should be accounted for. The agricultural sector plays an important role in achieving many of the goals, most obviously ‘zero hunger’ which cannot be achieved without food production, but also impacts on goals relating to the environment (Gil et al., 2018) such as ‘life on land’, ‘climate action’ and ‘end poverty’. Indeed, agricultural production systems have been identified as a major contributor to key global issues such as biodiversity loss, climate change and unsustainable nutrient cycling (Steffen et al., 2015; Burns et al., 2016; Campbell et al., 2017). This has led to increasing interest in understanding how agricultural production systems could be transformed to reduce negative environmental impacts whilst providing nutritious food and prosperous livelihoods within the sector (Kanter et al., 2018). Yet the complexity of these systems, their global scale and even their variability at local scale is a barrier to transformative change because it is difficult to identify alternatives to the current situation that take account of all the processes that might be affected by change and the multiple functions of agricultural landscapes.

One particular challenge is to stimulate informed stakeholder discussion about trade-offs within agricultural landscapes so that priorities can be identified collectively. This requires information about the likely trade-offs within agricultural systems and associated possibilities for managing these systems to meet different combinations of objectives. Various methods have been used for identifying trade-offs in agricultural systems, including participatory methods, empirical methods, the use of multi-objective algorithms with models of agricultural systems and combinations of the above (Klapwijk et al., 2014). Multi-objective algorithms are appealing because they can make use of the current understanding of systems that is embedded in models. They may need to be combined with other methods where key processes and objectives are not adequately represented in models.

Optimization algorithms strategically try different configurations of land management (the inputs to a model of an agricultural system) to identify an optimal value of a quantifiable objective or objectives (the outputs from the model). Multi-objective algorithms (e.g. Deb et al., 2002; Cao et al., 2011; Huang et al., 2013) are particularly useful because they avoid the need to weight different objectives (Kanter et al., 2018). Such approaches have been used to identify scenarios of land-use change between an agricultural use and a range of other uses (Polasky et al., 2008; Hu et al., 2015; Estes et al., 2016). In these, the spatial configuration of the relevant land-use categories is optimised using objectives such as agricultural production and environmental factors, including biodiversity and water retention. However, the different possible practices within each land-use category are not considered. Other studies, however, have also optimised the spatial configuration of agricultural land managed using different practices using a multi-objective approach (Groot et al., 2007; Zhang et al., 2012; Kennedy et al., 2016; Groot et al., 2018). Multi-objective algorithms therefore provide a useful way to explore the effect of both land use and management practices on different objectives simultaneously. Algorithms play a particularly interesting role in identifying possibilities because, whilst the objectives and search options are set by people, within this range the computer algorithm can search dispassionately and so consider options that might otherwise be discounted without due consideration due to preconceptions. For example, in a study focussed on land use possibilities in Iowa, Nassauer and Corry (2004) noted that whilst citizens might imagine future landscapes without perceiving unintended consequences, experts might limit their creativity based on what behavioural change they deem possible.

One challenge in using multi-objective algorithms is that the results are complex and can be difficult to interpret. If two objectives are considered and there is a trade-off between these two objectives, the multi-objective algorithm will identify a number of optimal points along a trade-off frontier. The points along this frontier have Pareto optimality, that is to say that at every point on the curve, an improvement in one objective would be associated with a negative effect on the other objective (see for example Lautenbach et al., 2013 for further explanation of Pareto optimality). Such results can be plotted easily on a 2-D plot (e.g. Zhang et al., 2012; Kennedy et al., 2016). If the algorithm considered three objectives, the Pareto frontier could be shown as a 3-D surface. However. as more objectives are included, the multi-dimensional surface becomes harder to plot and visualise. A variety of approaches have been considered to visualise results, including the use of different colours and sizes of points to represent additional dimensions and using heat maps (Lautenbach et al., 2013; Tušar and Filipič, 2015; Ibrahim et al., 2016). For high dimensions, however, it is intuitive to project the surface onto a series of 2-D plots representing the different pairs of dimensions (Groot et al., 2012). This allows the frontiers between each pair of objectives to be visualised. Still, such plots do not show the link from the land management actions to the associated outcomes (i.e. the associated point on the trade-off frontier). This can be done to a limited extent by illustrating a few key points, for example with a map of the land use that leads to a particular result (Polasky et al., 2008; Lautenbach et al., 2013). However, it is not possible to do this for a frontier with hundreds of points; thus, new approaches to enable this would aid interpretation of results.

Challenges in determining trade-offs within agricultural landscapes lie in the complexity of these systems, both in terms of the need to consider multiple functions of the system from economic, social and environmental perspectives and the need to consider different spatial scales. The spatial component of these systems is important to consider both because of the connectivity of landscape and landscape heterogeneity. The connectivity of the landscape means that altering a management practice in one location may directly affect contiguous locations due to physical flows (e.g. water, nutrients). Meanwhile the heterogeneity of landscapes means that actions taken to optimise objectives in one place may not be optimal in another (e.g. due to differences in soil types). However, this heterogeneity is also an opportunity, because different areas of land could be managed to take best advantage of their specific characteristics. This is the idea behind the concept of land sparing, the suggestion that environmental and food production might be best met by removing some land from agricultural production and using it to meet environmental objectives whilst increasing production on the land that remains in production (Phalan et al., 2011). Ultimately, to identify trade-offs in agricultural landscapes using multi-objective optimization, it would be desirable to use a single model that represents all relevant economic, social and environmental objectives as well as spatial variability and interactions. Such a model does not exist, but development of models and model frameworks that are able to represent multiple dimensions and spatial interactions in agricultural landscapes simultaneously is ongoing (van Ittersum et al., 2008; Schönhart et al., 2011; Groot et al., 2012; Schönhart et al., 2016). Meanwhile the Rothamsted Landscape model (Coleman et al., 2017) captures another part of this complexity. It focusses on agricultural production as well as the environmental component of agricultural landscapes, specifically simulating nitrous oxide emissions and leaching from the soil, allowing the spatial heterogeneity of the landscape to be considered.

In this paper the Rothamsted Landscape model (Coleman et al., 2017) is used to investigate and visualise trade-offs, using wheat production in the south east of the United Kingdom (UK) as an example. A specific aim is to consider the importance of spatial heterogeneity within the landscape, which we do by comparing trade-offs in three soil types (clay, sandy clay and sandy loam) and then identifying how the trade-offs change when these three soils are managed collectively, representing a small heterogeneous landscape. This includes management approaches in which some soils are managed for production objectives and others for environmental objectives within the search space for the multi-objective algorithm. The algorithm can then identify when objectives might be best achieved by sharing production and environmental objectives across sites and when they might be better achieved by reducing production at one site and maximising it at another to compensate, thus making use of landscape heterogeneity. The intention is that such results would be used to inform and stimulate stakeholder discussion, although we do not report the results of such an interaction here, focusing instead on the development of this modelling approach. We consider this as an illustrative example, with a relatively simple set of possible management possibilities that could be expanded in future work to further understand the importance of spatial heterogeneity and even landscape connectivity in managing trade-offs across the landscape. Using this example of wheat production in the UK, we develop a clustering approach to identify distinct management strategies and how these relate to different outcomes for the multiple optimization objectives. This aims to facilitate the interpretation of the results by associating possible land management strategies (i.e. similar types of management actions) with different regions of the trade-off curves. This helps to address the issue that for complex sets of objectives and land use and management options multi-objective algorithms can identify numerous possibilities which may become overwhelming.

## 2 Methods

### 2.1 Optimization algorithm

We coupled the Rothamsted Landscape model with an optimization algorithm to determine Pareto optimal fronts between multiple objectives defined in terms of outputs from the model as has been done previously (Coleman et al., 2017). The optimised Pareto fronts describe the synergies and trade-offs between objective variables such as crop yield and nitrous oxide emissions. In order to use such algorithms the user must define the optimization objectives and the control variables (in this case a number of different farm management actions). The algorithm varies the control variables and uses a simulation model (in this case the Rothamsted Landscape model) to calculate the effect of these controls on the objectives. The algorithm must be able to identify which sets of control variables result in better outcomes of the objectives and strategically identify new sets of control variables to try to see if even better outcomes can be achieved. NSGA-II (Deb et al., 2002) is an established algorithm to do this. Here, we combined the non-dominated sorting routine from NSGA-II with differential evolution (Storn and Price, 1997) to identify new sets of options to try. Differential evolution adds a directional component to the identification of new control variables which is useful for numerical control variables, as gradients can be used to inform the search direction. This approach is not relevant when the control variables are categorical and there is no ‘gradient’ between categories as they are distinctly different options. In this application, as the controls were numerical, the differential evolution approach was appropriate.

To run, the algorithm requires an initial list of management options to try; this forms the initial population of management strategies. This initial population can be formed by randomly selecting values for each of the management variables within each strategy. To do this a range or set of all the values possible for each management variable is defined. Alternatively, the initial population could be based on management strategies that are of interest, perhaps because they represent current practice or an extreme management option. Here, the initial population was predominantly random but was also seeded with some strategies representing current practice and extremes.

The algorithm then implements each of the management strategies from the initial population in the simulation model and records the effect on each of the multiple objectives. Non-dominated sorting then identifies the management options that result in the ‘best’ objectives, i.e. those that are non-dominated. A point is said to be dominated by another if it is worse for every single objective.

The process is iterated in directions that the differential evolution algorithm suggests will be an improvement, until the results converge and produce a similar Pareto front with each iteration. The algorithm was run for 1500 iterations and convergence was judged manually by visually comparing the frontier over multiple iterations. Running the algorithm for this application took around 1–2 days for each soil, although the time depends on the control variables that are chosen as some combinations take longer to run than others.

When considering the management of multiple units of land with different characteristics and management possibilities, there was also a second stage of optimization to combine the three frontiers across the land uses. This used the pareto fronts generated for each soil using the simulation model as an input to the NSGA-II multi-objective optimization algorithm algorithm (i.e. without differential evolution being implemented, as the control variables are categorical and a directional search is not helpful in this context). By using the pareto frontiers identified in the first step (i.e. the sets of points identified for each soil), we assumed that there were no interactions between the sites and that what was optimal at one site was not affected by actions at other sites. The algorithm was then used to consider how three sites with known individual trade-off curves could be managed together to produce the best average values of the objectives. There was one control variable for each unit of land, this control variable was an index value identifying the point on the trade-off curve for that site. As the optimal trade-off curves for each soil consisted of 100 points, there are a million possible combinations of management practices. The algorithm thus effectively searches for the best way in which the trade-off curves from different locations could be combined by taking into account the strengths of each location and where they can best contribute to specific objectives. A genetic population of 1000 points was used in this search, primarily to better represent the resulting trade-off frontier as the shape of the surface becomes more complex.

### 2.2 Simulation model scenario

The optimization algorithm used outputs from the Rothamsted Landscape model (Coleman et al., 2017) to simulate the effect of the management options described by the control variables on the objectives. This model has been calibrated and validated in South-East England, within the climatic zone of the study, (Coleman et al., 2017). It operates at a daily time step and simulates agricultural yield as well as the effect of production on environmental processes including nutrient leaching and nitrous oxide emissions. The Landscape model is also able to simulate nutrient flows across the landscape, however this feature of the model was not used here. Instead, the model was used to simulate the trade-offs between multiple objectives at a single location at a time.

The model was used to simulate wheat production using weather data that represents conditions in the climatic zones in the west and centre of England. To do this weather data from Chivenor, Devon, was used. The simulations were initialised with soil textural data representing Clay, Sand Clay and Sandy-Loam soils (Table 1).

**Table 1:** Soil properties (0–23 cm) of the three soil types used in simulations.

	Clay	Sandy Clay	Sandy Loam
Clay (%)	76	36	14
Silt (%)	14	15	18
Sand (%)	10	49	68
SOC (%)	2.49	1.83	0.96
pH	7.63	7.14	6.03
Bulk density	1.23	1.38	1.33

In the second stage of the paper, when combined managements of the soils were considered, equal areas of each of the three soil types were assumed. Thus the objectives were quantified by taking the arithmetic means of the values at each site.

### 2.3 Control variables

The identification and implementation of appropriate control variables is critical as it sets the range of possibilities that the optimization algorithm can explore. Whilst it is therefore tempting to make the scope wide, this can slow down the optimization algorithm or prevent it from finding global optima. Here, we used 11 control variables – the first 9 of these represented the amounts of ammonium nitrate fertiliser applications. Each application could vary between 0 and 100 kgN/ha. The first application can be made on 1st March with possible subsequent applications at 2 week intervals. If it rained on the day that any application was scheduled, that application was delayed until the next day. The expectation here, was that several of the possible 9 applications would be 0. If the initial values for these application rates were drawn from a uniform distribution it would be highly unlikely that the value zero would be selected repeatedly. Thus, to improve the convergence of the algorithm, up to half of the initial population was set to include members that had 6–8 zero values for N application control variables whilst the remaining members of the population had 9 randomly sampled application rates. The 10th control variable was a farm yard manure (FYM) application (0–3 t/ha) and the 11th control variable determined the time at which this manure application was applied from 0 to 3 weeks before sowing.

### 2.4 Objectives

The optimization objectives were selected to represent indicators that are relevant to the contribution of agriculture to the SDGs, either directly by production or due to the effects of production on the surrounding environment. A number of possible SDG indicators for agriculture have been proposed (Gil et al., 2018), here however, we focused on those for which it was possible to quantify with the model. These were; crop yield, nitrogen use efficiency (NUE), nitrogen surplus, nitrous oxide emissions, and change in soil organic carbon (SOC). The yield and nitrous oxide emissions were simulated for each year and then calculated as the average over the nine seasons of the simulation. The change in SOC was calculated as the difference between the value at the start and the end of the simulation. These values are clearly sensitive to the initial SOC. The NUE and nitrogen surplus objectives were calculated by first summing the inputs and outputs in the crop grain and straw over the whole simulation. The NUE was then calculated as the ratio between the outputs and inputs, and the surplus as the difference between inputs and outputs. All sources of nitrogen entering the soil were accounted for, so including atmospheric deposition (Coleman et al., 2017).

In addition, a profit function is calculated, as the sum of the yield each year multiplied by the farm price of the crop, minus the total cost of the N fertiliser applied (both the mineral N and N in FYM), minus the total cost of the P fertiliser applied, minus the cost of applying the N fertiliser. This is divided by the number of years to give the average profit.

### 2.5 Clustering

To identify common management strategies, the sets of control variables found to be optimal were further analysed using a cluster analysis. Prior to clustering, the nine inorganic fertiliser application values were summarised into 3 values; the total amount of N applied, the number of N applications (i.e. number of non-zero values), and the timing of the first application. The cluster analysis was then performed on sets of variables representing the three values summarising nitrogen fertiliser and the amount of FYM applied. The cluster analysis used a minimum variance, hierarchical clustering approach following the Ward (1963) method. This was implemented in MATLAB (MATLAB and Statistics Toolbox Release, 2018) using the standardised Euclidean distance. To aid visualisation, the mean profitability factor for each cluster was calculated, and only the most profitable strategies were highlighted in the trade-off curves.

## 3 Results

**Fig. 1:**
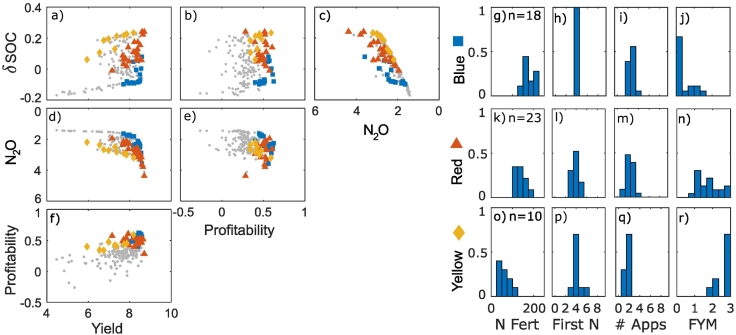
Trade-off frontiers (a-f) and cluster characteristics (g-r) in clay soil. Units are: Yield (t/ha), Profitability (x10^3^ £ / ha/year), N_2_O (x10^3^ CO_2_ equivalent yr^−1^), change in SOC - δSOC (%). Note that for N_2_O, increasing values are shown from right to left or top to bottom because this objective was minimised in the optimization process. This means that, consistently across the plots, trade-offs show trends from the top left to the bottom right of the plots and synergies trends from the bottom left to the top right. Points within the most profitable clusters are highlighted; all other points are shown as small grey circles. Histograms of the cluster variates show the fraction of the points in each cluster with a particular management value, where n is the number of management strategies (i.e. points) in each cluster, N Fert is the total N applied in fertiliser, First N is the week of the first N application, # Apps is the number of fertiliser applications and FYM is the amount of farm yard manure applied.

**Fig. 2:**
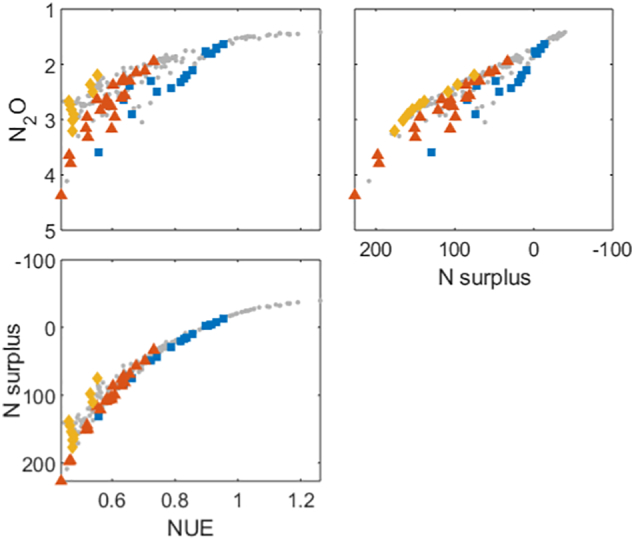
Trade off frontiers between the objectives relating to nitrogen cycling for the clay soil. Units are: N_2_O (x10^3^ CO_2_ equivalent yr^−1^), N surplus (kg ha^−1^ yr^−1^), NUE (−). Yellow diamonds, red triangle and blue squares indicate points in the frontier that correspond to the clusters detailed in Fig. 1, g-r grey dots show all other points in the frontier. Note that for N_2_O and N surplus, increasing values are shown from right to left or top to bottom because these objectives were minimised in the optimization process. This means that, consistently across the plots, trade-offs show trends from the top left to the bottom right of the plots and synergies trends from the bottom left to the top right.

The optimization approach identified trade-off frontiers between the different objectives. Scatter appears in the frontiers because the plots shown a multi-dimensional surface projected onto a 2-D plot. Trade-offs occur when an improvement in one objective has a detrimental effect on another objective. Meanwhile synergies occur when objectives improve concurrently. In the clay soil, this approach identified trade-offs between the yield and N_2_O emissions and the N_2_O emissions and the change in SOC (Fig. 1a–f). As there were synergies between the N_2_O emissions, NUE and the N surplus (Fig. 2), the trade-offs between NUE and N surplus indicators with other objectives were the same as those for the N_2_O objective (Supplementary information, Fig. S2). Meanwhile synergies were observed between the yield and profitability, yield and change in SOC, and the profitability and the change in SOC. It is instructive to focus on the N_2_O data because these results emphasise the non-linearity of certain trade-offs (Fig. 1d). Specifically, the line that would represent the 2-D frontier between N_2_O and each of the other objectives is non-linear. The frontier between the profitability and the N_2_O emissions suggests a synergy at high emission values and a trade-off at lower emission values (Fig. 1e), however there is also a lot of scatter behind the frontier corresponding to the other objectives. As such, to meet these other objectives it may not be desirable to optimise the N_2_O emissions per unit profit.

**Fig. 3:**
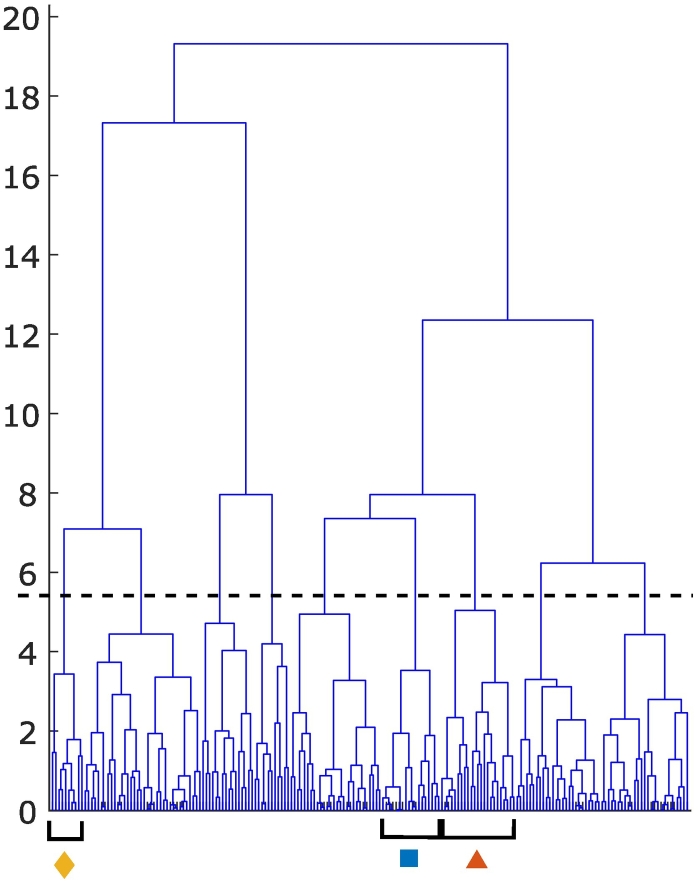
Hierarchical cluster results for clay soil, with the profitable clusters highlighted. The dotted line indicates the division of the dataset into 9 clusters.

The cluster analysis approach was applied to look for similarities in the control variables from within the optimal population identified by the optimization algorithm. We refer to these clusters as ‘management strategies’ as they group together similar sets of management actions allowing them to be associated with their effect on the objectives. For the clay soil, hierarchical clustering was used to divide the sets of management actions into 9 clusters (Fig. 3). Looking at the mean of the profitability objective in the clusters, three profitable strategies were identified:

1. Applying no FYM and relatively high fertiliser N over 3 applications
2. Applying a little FYM and a slightly less N fertiliser over 2 applications
3. Applying much FYM and rather less N fertiliser over 2 applications

Notably, in these strategies, fertiliser applications tended to start later (from the 4th possible application date) than other less-profitable strategies (Supplementary information, Fig. S1). The first two strategies were associated with high yield, whilst for the third profitable strategy the yield was slightly less and the profitability arose from lower fertiliser costs. FYM application was, unsurprisingly, associated with increases in SOC. Most of the profitable strategies were associated with high N_2_O emissions, except for a subset of the first strategy (when no FYM and lower amounts of N fertiliser were applied). Also, in the clay soil the maximum yield was higher than the sandy clay and sandy loam soils.

**Fig. 4:**
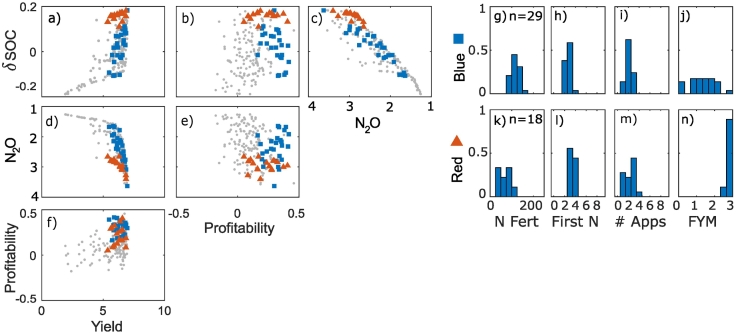
Trade-off frontiers (1-f) and cluster characteristics (g-r) in sandy clay soil. Units are: Yield (t/ha), Profitability (x10^3^3 £/ha/year), N_2_O (x10^3^ CO_2_ equivalent), Change in SOC - δSOC (%). Note that for N_2_O, increasing values are shown from right to left or top to bottom because this objective was minimised in the optimization process. This means that, consistently across the plots, trade-offs show trends from the top left to the bottom right of the plots and synergies trends from the bottom left to the top right. Points within the most profitable clusters are highlighted; all other points are shown as small grey circles. Histograms of the cluster variates show the fraction of the points in each cluster with a particular management value, where n is the number of management strategies (i.e. points) in each cluster, N Fert is the total N applied in fertiliser, First N is the week of the first N application, # Apps is the number of fertiliser applications and FYM is the amount of farm yard manure applied.

In the sandy clay soil the same trade-offs and synergies between objectives were observed as in the clay soil (Fig. 4a–f). Two profitable management strategies were identified (Fig. 4g–n):

1. High application of FYM, 1–3 applications of a small amount of N fertiliser
2. Low or Medium application of FYM, 1–3 applications of a medium amount of N fertiliser

In this soil, most of the points in the optimal set were high yielding (Fig. 4f). Profitable strategies were also associated with high yields. The greatest possible profitability was less than in the clay soil.

**Fig. 5:**
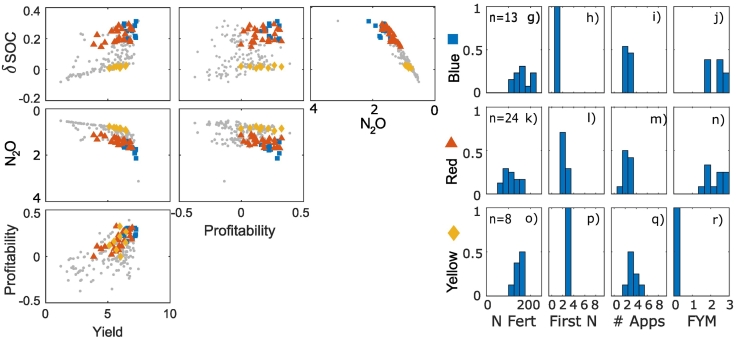
Trade-off frontiers (1-f) and cluster characteristics (g-r) in sandy loam soil. Units are: Yield (t/ha), Profitability (x10^3^ £ / ha / year), N_2_O (x10^3^ CO_2_ equivalent), Change in SOC - δSOC (%). Note that for N_2_O, increasing values are shown from right to left or top to bottom because this objective was minimised in the optimization process. This means that, consistently across the plots, trade-offs show trends from the top left to the bottom right of the plots and synergies trends from the bottom left to the top right. Points within the most profitable clusters are highlighted; all other points are shown as small grey circles. Histograms of the cluster variates show the fraction of the points in each cluster with a particular management value, where n is the number of management strategies (i.e. points) in each cluster, N Fert is the total N applied in fertiliser, First N is the week of the first N application, # Apps is the number of fertiliser applications and FYM is the amount of farm yard manure applied.

For the sandy loam soil (Fig. 5), the highest possible profitability was £390 ha^−1^ yr^−1^, lower than the sandy clay and clay soils (£458 and £624 ha^−1^ yr^−1^ respectively). Meanwhile, the maximum possible yield 7.5 t/ha for the sandy loam soil, was higher than possible for the sand clay (7.0 t/ha) but lower than for the clay soil (8.7 t/ha).

The 3 profitable strategies identified were:

1. High FYM, high N fertiliser in 2–3 applications, starting early
2. High FYM, medium N fertiliser, typically 2–3 applications, starting slightly later
3. No FYM, medium N fertiliser, typically 3–4 applications, starting even later

**Fig. 6:**
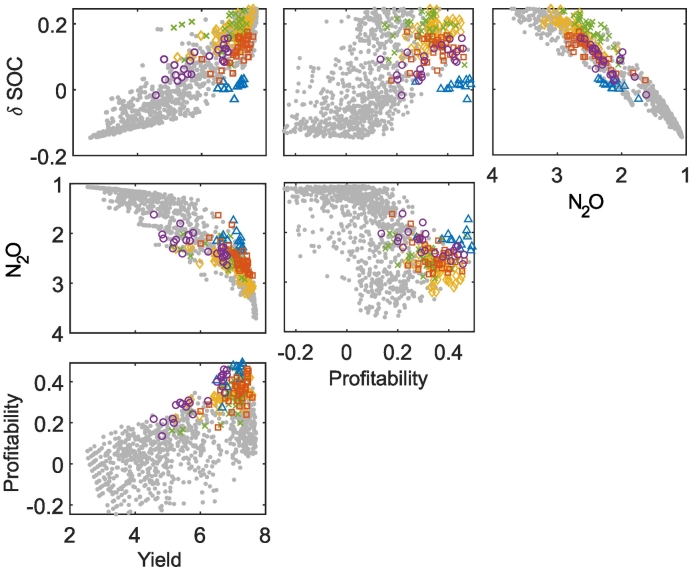
Trade-off frontiers when managing three fields each of equal area but each of a different soil texture (clay, sandy clay, sandy loam). Units are: Yield (t/ha), Profitability (x10^3^ £ / ha / year), N_2_O (x10^3^ CO_2_ equivalent), Change in SOC - δSOC (%). Note that for N_2_O, increasing values are shown from right to left or top to bottom because this objective was minimised in the optimization process. This means that, consistently across the plots, trade-offs show trends from the top left to the bottom right of the plots and synergies trends from the bottom left to the top right. Points within the most profitable clusters are highlighted; all other points are shown as small grey circles. Histograms of the cluster characteristics for the most profitable clusters are shown in Fig. 7.

Combining the objectives across three fields of equal area but differing in soil texture (clay, sandy clay, sandy loam) led to a combined trade-off frontier (Fig. 6). Notably, for the combined management, it becomes more clear that the frontier is a multi-dimensional surface with the Pareto optimal points more spread out compared to the management for each of the soils individually (Fig. 1, Fig. 4, Fig. 5). The relationship between the profitability and N_2_O emissions was synergistic for high emissions, but becomes a trade-off at lower emissions. This change at the frontier, from synergy to trade-off, was clearer than in the individual soils and indicated that a reduction in nitrous oxide emissions beyond a certain point would be associated with a large reduction in profitability (Fig. 6e). Multiple profitable strategies perform similarly with respect to multiple objectives (e.g. green and yellow clusters in Fig. 6) meaning that there is freedom to make choices between these strategies based on additional objectives not captured by the model. The most profitable strategies (the blue cluster in Fig. 6) produced lower nitrous oxide emissions than the other profitable clusters, all of which resulted in an increase in SOC.

**Fig. 7:**
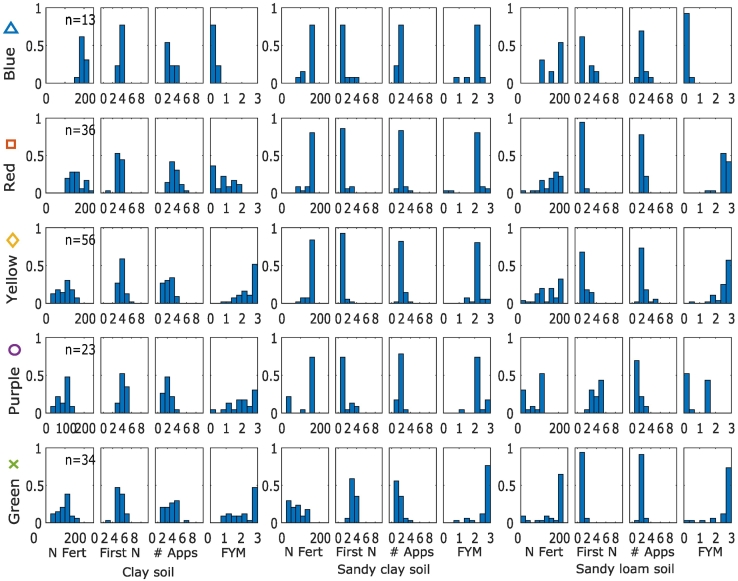
Characteristics of the profitable clusters for the combined management of three soil types. Histograms of the cluster variates show the fraction of the points in each cluster with a particular management value, where n is the number of management strategies (i.e. points) in each cluster, N Fert is the total N applied in fertiliser, First N is the week of the first N application, # Apps is the number of fertiliser applications and FYM is the amount of farm yard manure applied.

**Fig. 8:**
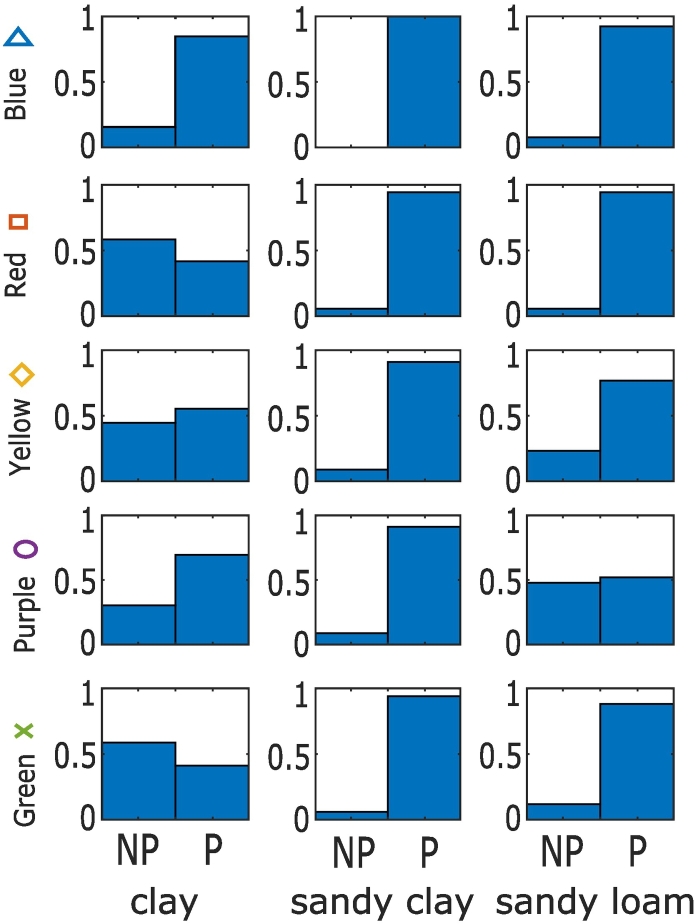
Proportion of the points within each cluster that correspond to profitable management of that soil type. On the x-axis, P corresponds to profitable and NP to not profitable. Profitable points are defined as the 30% of most profitable points for that soil type.

Interestingly, all of the more profitable clusters in the combined management included the most profitable management strategies for the sandy clay soil, which had the lowest maximum yield (Fig. 8). Furthermore, only one of the five management strategies on the sandy loam soil (which had the medium maximum yield) included less profitable management strategies on this soil. On the clay soil (which had the highest maximum yield), there were a wider range of management strategies that resulted in profitable overall management. Compared to the strategies identified for the clay soil alone (Fig. 1.), the strategies that were profitable on clay in the case of combined management included more in which manure was applied to the clay soil, and more numerous fertiliser applications (Fig. 7).

This shift to less profitable strategies occurs in the clay soil but not in the sandy clay when combined management is considered. The reasons for this are complex but occur because of the effect that a decrease in profitability has on the other objectives in each of the soils. For example, in the sandy clay soil, most of the possible reduction in N_2_O emissions can be achieved whilst remaining profitable (i.e. the frontier has a fairly straight vertical edge in Fig. 4e). In the clay soil however, there is a discernible trade-off that emerges to reach the lowest possible emissions (i.e. the top edge of the frontier is more rounded in Fig. 1e). Additionally, in the clay soil the differences between profitability of the more profitable strategies was smaller than in the sandy clay soil (i.e. with respect to profitability, the points are grouped together predominantly in the high profitability region for the clay soil – Fig. 1, but are more spread out for the sandy clay soil – Fig. 4). This means that, to maintain overall profitability for a decrease in the profitability in the sandy clay soil, a relatively large increase in profitability on another soil would be necessary. Hence optimal combined strategies maintain profit in the sandy clay soil.

## 4 Discussion

### 4.1 Trade-offs between objectives

One distinctive feature of the results is the non-linearity of the trade-off between yield and N_2_O emissions. This is not unexpected as high yields are associated with high N application, either in the form of fertiliser or manure, but is important to note because many national greenhouse gas inventories follow an emissions factor approach that effectively assumes this relationship is linear (Eggleston et al., 2006; Shcherbak et al., 2014). Recent work has suggested that the increase in N_2_O emissions with increasing N application is non-linear (Shcherbak et al., 2014), and here, when the trade-off is considered with respect to yield, this non-linearity is exacerbated as at high N application further increases in N applied result in only a marginal increase in yield. Linquist et al. (2012) considered the trade-off between greenhouse gas and cereal crop production. They concluded that the lowest global warming potential per unit yield occurred at 91% of potential yield for wheat. This is comparable to the point at which we observed the non-linear increase in GHG production (90% of the maximum yield in the clay soil, 85% in the sandy clay and 88% in the sandy loam soil). A similar finding was also reported by Nguyen et al. (2018) who suggested that 90% of potential production can be achieved with minimal impacts, including greenhouse gas emissions. It is interesting to note that this non-linear point is comparable to the 80% of potential yield that is often considered as the ‘exploitable yield’ in yield gap analysis (Lobell et al., 2009; van Ittersum et al., 2013). Whilst this exploitable yield has been reached from a resource use and profitability perspective, in our analysis it corresponds more to the threshold that limits a negative environmental impact, as some of these high emission management strategies still appear to be profitable in our analysis.

The results also highlight a trade-off between N_2_O emissions and increasing SOC in the soil. This is in part due to the control options by which the SOC can be increased in the simulations; either by manure addition or by increasing N application in such a way that yield increased and hence crop residues also. Both mechanisms are typically associated with an increase in N_2_O emissions. Bos et al. (2017) showed that net GHG emission reductions could not be obtained with manure application, and only the application of compost resulted in larger emission reductions because of SOC increase compared to N_2_O emission increase. There are also indications that N_2_O emissions may be inherently higher from soils with higher SOC (Palmer et al., 2017; Charles et al., 2017), as this would reduce the chance that N_2_O emissions are limited by C availability in soil (Charles et al., 2017). Other studies, however, have not found a significant effect of SOC (Buckingham et al., 2014). When considering carbon sequestration to mitigate GHG production, the net effect of sequestration and emissions must be considered. Other studies have also suggested that, in terms of global warming potential carbon sequestration may be offset by N_2_O emissions (Powlson et al., 2011; Zhou et al., 2017). A systems perspective is also clearly necessary as, if manure was not applied to the soil, it would still emit greenhouse gases elsewhere (Hou et al., 2015). However, increases in SOC are also desirable for other reasons such as increasing future soil fertility (Garratt et al., 2018) and reducing erosion risk.

The EU nitrogen panel has made recommendations for NUE and N surplus (EU Nitrogen Expert Panel., 2015), suggesting a range of NUE from 0.5 to 0.9 combined with an N surplus of less than 80 kg ha^−1^ yr^−1^. In our study these ranges were met by using strategies in which very little manure was applied, for example the blue square cluster (Fig. 2) in which no manure was applied or very few of the red triangle cluster in which a small amount of manure was applied. This corresponds to the fact that manure applications were associated with an increase in N_2_O emissions in the simulations, and also with increased N leaching. Some thought must be given to how NUE and N surplus is calculated when applying organic matter to the soil, as nitrogen applied in one year may benefit crops in future years. For this reason N inputs and outputs were calculated for the whole simulation, however, there was also likely a build up of soil nitrogen in this period. Indeed, an increase in SOC would require this (Van Groenigen et al., 2017); So if increasing SOC is an objective, the NUE and N surplus targets or calculation approaches may need to be reconsidered concurrently.

### 4.2 Management across soil types

As expected, the model simulated differences between the soils in terms of the yield and N_2_O emissions. The N_2_O emissions from the sandy loam soil were notably less than from the other two soils, particularly at greater N application rates. This corresponds to the findings from other studies which suggest that emissions from fine textured soils are greater than from coarse textures (Charles et al., 2017) and that water filled pore space is a key factor affecting emissions (García-Marco et al., 2014). Thus soils which retained more water, emitted more N_2_O. The lower NUE values and higher N surplus values in this soil also suggest that more N is lost from the soil profile by leaching, as would be expected.

Interestingly, the maximum possible increase in SOC was comparable for all three soils. The simulated potential is of course related to the initial SOC in the simulations. In this case, the simulations were based on soils under long-term arable management with low initial SOC and in this situation it seems that, for the soil textures we considered, the soil texture had little effect on the possible increase in SOC.

In general, profitable strategies were associated with large yields. At the highest yields, we might have expected to see a trade-off between these two objectives; indeed, in yield gap analysis, 80% of the potential yield is considered as the ‘exploitable yield’ (Lobell et al., 2009; van Ittersum et al., 2013), representing a point at which there starts to be a trade-off between the two objectives because the cost of inputs outweighs the increase in sale price due to the increase in yield. Here, however, as in other studies (Silva et al., 2017), we did not observe this trade-off. Nevertheless, in the clay soil, a reduction of yield to around 70% of the maximum could be achieved with very little impact on the profitability (Fig. 1). In the sandy clay, reductions in yield resulted in a linear reduction in profitability (Fig. 4). In both case, strategies that reduced yield but maintained profitability were associated with large manure application rates and small amounts of N fertiliser. This strategy was not identified as a possibility in the sandy clay soil, which also had the lowest yield potential. In this soil, all the profitable strategies were associated with high yields. This suggests that there is less opportunity to adapt management strategies whilst remaining profitable.

On many farms, such as those in the UK, different soil types are present and these soils must be managed simultaneously. Considering the combined options across these different soils means that additional strategies can be identified to deliver the same objectives overall. Here, for example, the clay soil could be managed in a way that was not necessarily the most profitable for that soil, but contributed to improving the other objectives. The loss in the likely profit from this soil could then be compensated for on another soil in which the other objectives were less desirable.

One ongoing debate regarding sustainable agriculture relates to the notion of sharing or sparing land in agricultural production (Phalan et al., 2011; Fischer et al., 2014). This relates to considering whether environmental objectives might be best achieved by reducing production (and thus negative environmental impacts) across all agricultural land (sharing) or whether it would be preferable to remove some agricultural land from production entirely and use remaining agricultural land even more intensively (sparing). Most research in this area has focussed on the trade-off between biodiversity and production. However, recent studies suggest that land-sparing might help mitigate leaching and GHG emissions as well (Lamb et al., 2016; Balmford et al., 2018).

Generally, for land sparing it might be expected that the least productive agricultural land be removed from production because the focus is on yield and profit. Contrary to this, however, the results here suggest that it is management of the most productive soil that should be targeted to improve the multi-objective performance. Biodiversity is not a part of this analysis, nor is carbon sequestration in spared land. In this study, land cannot be entirely removed from production in the way the control variables in this study have been implemented, and the environmental objectives focus mainly on nitrogen. However, given the typical nitrogen response curve of crops, it is unsurprising that spreading nitrogen thinly over a larger area will be preferable to putting the same amount on a smaller area. This takes advantage of the larger yield increase per unit nitrogen that occurs at low application rates compared to those that occur at higher application rates. This means, however, that in a land sparing scenario, the agricultural land that is managed more intensively is likely to result in higher nitrous oxide emissions per unit yield. A natural extension of this work would therefore be to include biodiversity objectives within the optimization. Any trade-off between these objectives and nitrogen cycling objectives should then become apparent.

### 4.3 Envisioning future landscapes

One core aim of this paper is to illustrate the potential of of this approach to identify possibilities for possible strategies for managing agricultural landscapes. Notably the approach identifies many possibilities, the intention being that these can be presented to and discussed with stakeholders. Specifically, this approach could be used as a tool within a visioning and backcasting exercise (i.e. envisioning the future and then working backwards from this vision until the current state is reached). Visioning and backcasting is an approach that was developed in the energy sector as a tool to identify transformation pathways (Robinson, 1982) and has subsequently been used in other sectors as a tool for considering transformative change within complex systems (Dreborg, 1996; Vergragt and Quist, 2011). The first step is to envisage a desirable future and this is often done using a participatory that brings together multiple stakeholders with different perspectives. The idea in the visioning step is to focus on the key factors that are important to the different stakeholders for the future, rather than discussing the current problems and barriers to change (as can easily happen in a forecasting approach, or when the current situation is the focus of discussion). Gil et al. (2018) represent an example, where priorities for SDG-2 (‘End Hunger’) were set by comparing SDG-2 indicator target values for 2030 with current values. With the end vision in sight, the backcasting process then allows stakeholders to be more creative in considering how any barriers might be overcome. Thus, in theory, the approach should allow more room for a truly transformative pathway to be identified.

Such approaches encourage idealism, the philosophy being that our visions provide the motivation to develop new approaches and therefore reshape what is possible (Wright, 2010). Yet this idealism must be balanced with realism in order to generate visions that are also plausible and tangible so that action can be taken (Wiek and Iwaniec, 2014). Without this, there is a risk that an idealistic future vision may include multiple objectives that are not physically possible to achieve concurrently. Trade-off frontiers identified by multi-objective algorithms could therefore be used as a tool to encourage stakeholders to discuss trade-offs whilst they are developing this future vision. This would allow challenging discussions about trade-offs to occur during the visioning process, rather than during the backcasting process, thus with less focus on challenges that occur within the current system and more focus on what would be desirable in future. For instance, the example presented in this study could be used to inform stakeholder discussion about the relative importance of minimising nitrous oxide emissions from soil compared to maximising yield, without the idealistic assumption that both can be achieved simultaneously and without apportioning blame with regards to the current state of the system.

In this example the focus was on wheat production in a small landscape and with a defined set of control variables relating to fertiliser and manure application. Thus, there is clearly scope to expand the method to consider more diverse agricultural practices in more complex landscapes as the scale and context will affect the trade-offs that can be achieved. These could include practices that are of interest to stakeholders within a particular context and things that are technically possible and the scales (field, farm, region) at which different options could be implemented. Even so, the approach highlighted the range of possibilities that might be achievable with simple changes and the opportunities in considering the heterogeneity of the landscape.

There are various technical challenges in the optimization approach, including the risk of the algorithm becoming stuck in local minima and the inconvenience of the algorithm converging slowly because extreme control variables are selected and are difficult to simulate. These risks would be even more present in more complex modelling scenarios considering more complex landscapes and management possibilities. However, we found that seeding some of the initial population with a number of likely scenarios was effective at reducing the number of steps needed for convergence, an approach that has been useful elsewhere (Milne et al., n.d.). Within the initial population, several possible fertiliser applications were set to a rate of zero. When using more complex sets of control variables, subgroups of these control could be optimised initially in order to be able to seed optimization of the complete set.

The cluster analysis was used as a tool to relate the control variables to the resulting sets of objectives. This allowed the management strategies to be associated with different regions of the Pareto front. It is particularly interesting that for some pairs of objectives similar trade-offs can be achieved by alternative strategies (e.g. those from different clusters). For example, similar yields and GHG emissions occur for strategies identified by the clusters of red squares and green crosses in Fig. 6. In this case, more points occur in the red square cluster and fewer in green cross cluster. In general, the points in the green cross cluster dominate those in the red square cluster with respect to the indicators of SOC. Without this dominance in another factor, it is likely that this management strategy would occur less frequently in the population. Indeed, if the algorithm were optimised based on yield and GHG objectives alone it may even be overlooked entirely, if a strategy from the red square cluster marginally outperforms the strategy of the green cross cluster. Thus inclusion of another objective enabled the identification of an alternative management approach with similar performance for another objective.

To capture the complexity and the multiple stakeholder objectives and to identify a diverse range of strategies, it may seem desirable to include more objectives. However, with more objectives it becomes increasingly difficult to visualise the results and communicate them clearly. Additionally, models are unlikely to be able to simulate all of stakeholders' priorities. We suggest, therefore, that the objectives simulated and optimised by the model are viewed as a subset of the stakeholders' priorities. In this case the objectives used in the model were a subset of those identified by Gil et al. (2018) which included NUE, N surplus and greenhouse gas emissions intensity as priorities for agriculture in the Netherlands as well as pesticide use and genetic diversity which cannot currently be represented in the model. In most situations there will also exist additional objectives that have not been quantified by the model which stakeholders will be considering when they interpret the results. For each management strategy, another analysis exercise (e.g. a participatory method with the stakeholders or empirical evidence) could then be used to identify how the management strategies would likely affect unmodelled objectives. However, there may also be distinct management strategies that are appealing to stakeholders that exist close to the frontier but are neglected by the optimization algorithm. Although it is likely to increase convergence time, future algorithms should retain solutions that are ‘almost’ optimal within a chosen tolerance, particularly if they are associated with distinctly different management strategies. Given complex control variables, this should increase the number of distinct management strategies that might be of interest for stakeholder discussion and might meet other, untested objectives such as those relating to biodiversity.

## Appendix A Supplementary data

Cluster characteristics and trade-off frontiers for all clusters, including less profitable ones. Image 1
