# Supplementary material for: Multi-objective optimization as a tool to identify possibilities for future agricultural landscapes
Source: Sci Total Environ. 2019 Oct 15;687:535–45. doi: 10.1016/j.scitotenv.2019.06.070 (PMC6692559; doi:10.1016/j.scitotenv.2019.06.070)
Supplement: Supplementary file 1 — Cluster characteristics and trade-off frontiers for all clusters, including less profitable ones. [file mmc1.pptx]

## Slide 1
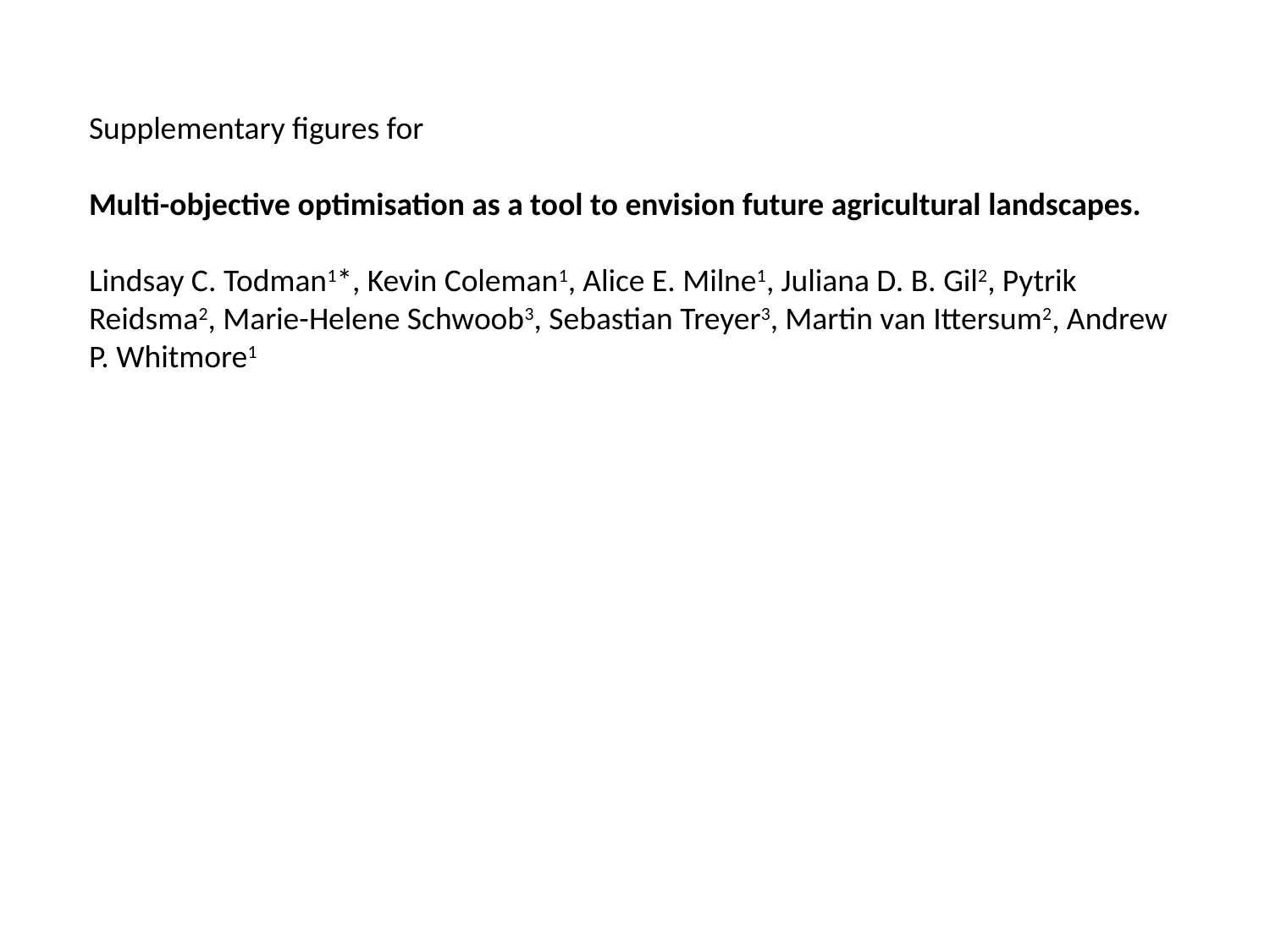

Supplementary figures for
Multi-objective optimisation as a tool to envision future agricultural landscapes.
Lindsay C. Todman1*, Kevin Coleman1, Alice E. Milne1, Juliana D. B. Gil2, Pytrik Reidsma2, Marie-Helene Schwoob3, Sebastian Treyer3, Martin van Ittersum2, Andrew P. Whitmore1

## Slide 2
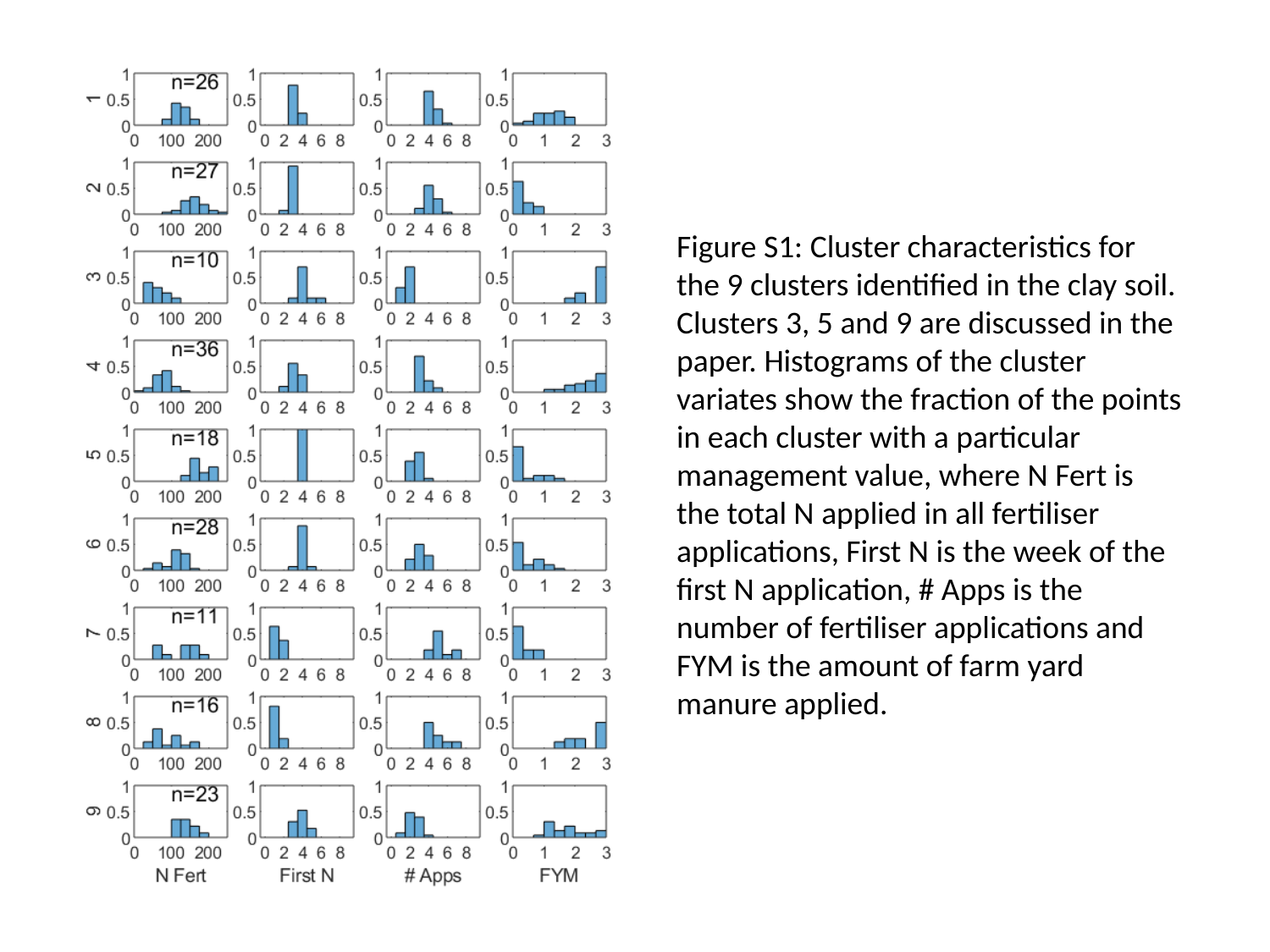

Figure S1: Cluster characteristics for the 9 clusters identified in the clay soil. Clusters 3, 5 and 9 are discussed in the paper. Histograms of the cluster variates show the fraction of the points in each cluster with a particular management value, where N Fert is the total N applied in all fertiliser applications, First N is the week of the first N application, # Apps is the number of fertiliser applications and FYM is the amount of farm yard manure applied.

## Slide 3
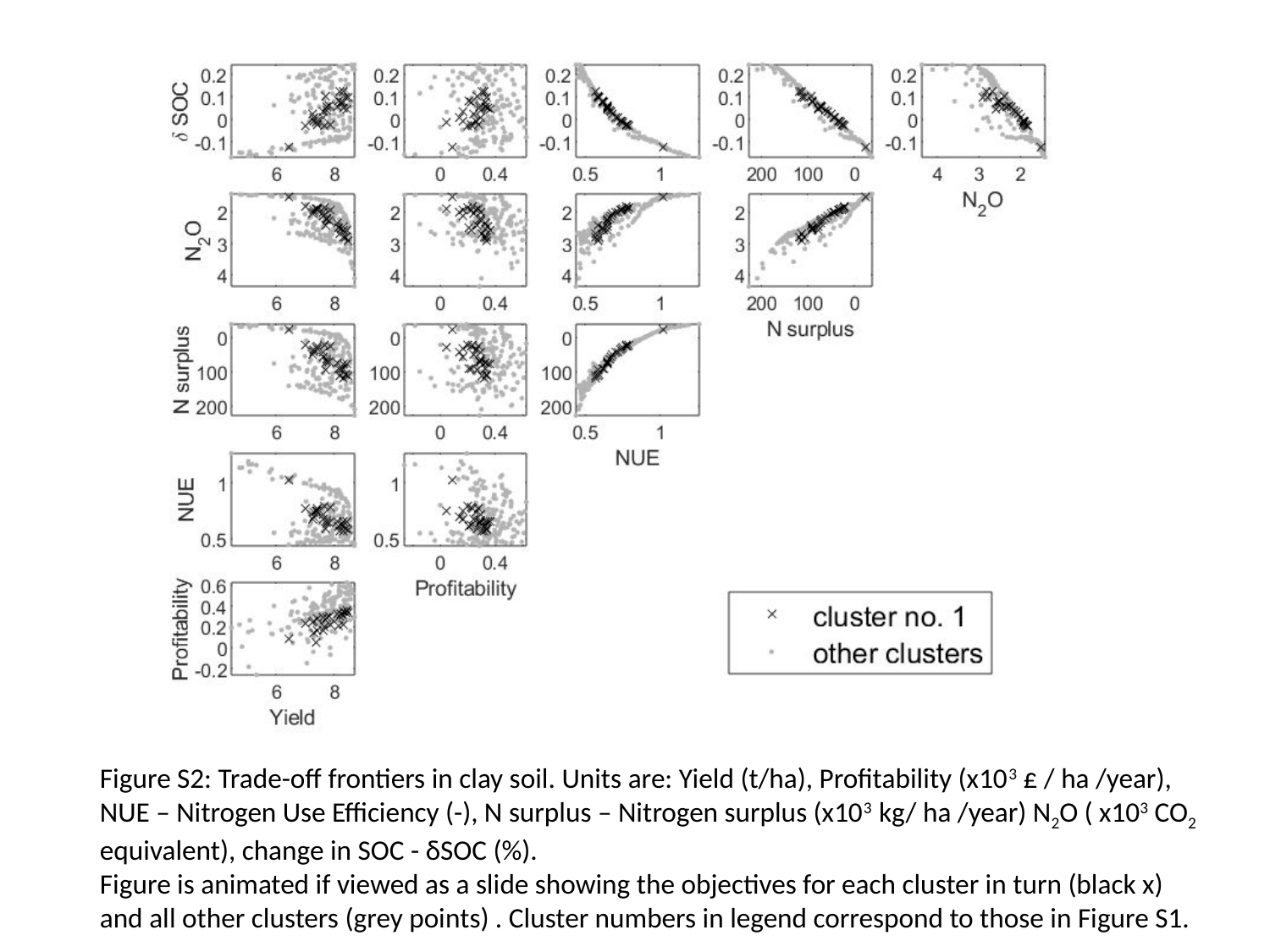

Figure S2: Trade-off frontiers in clay soil. Units are: Yield (t/ha), Profitability (x103 £ / ha /year), NUE – Nitrogen Use Efficiency (-), N surplus – Nitrogen surplus (x103 kg/ ha /year) N2O ( x103 CO2 equivalent), change in SOC - δSOC (%).
Figure is animated if viewed as a slide showing the objectives for each cluster in turn (black x) and all other clusters (grey points) . Cluster numbers in legend correspond to those in Figure S1.

## Slide 4
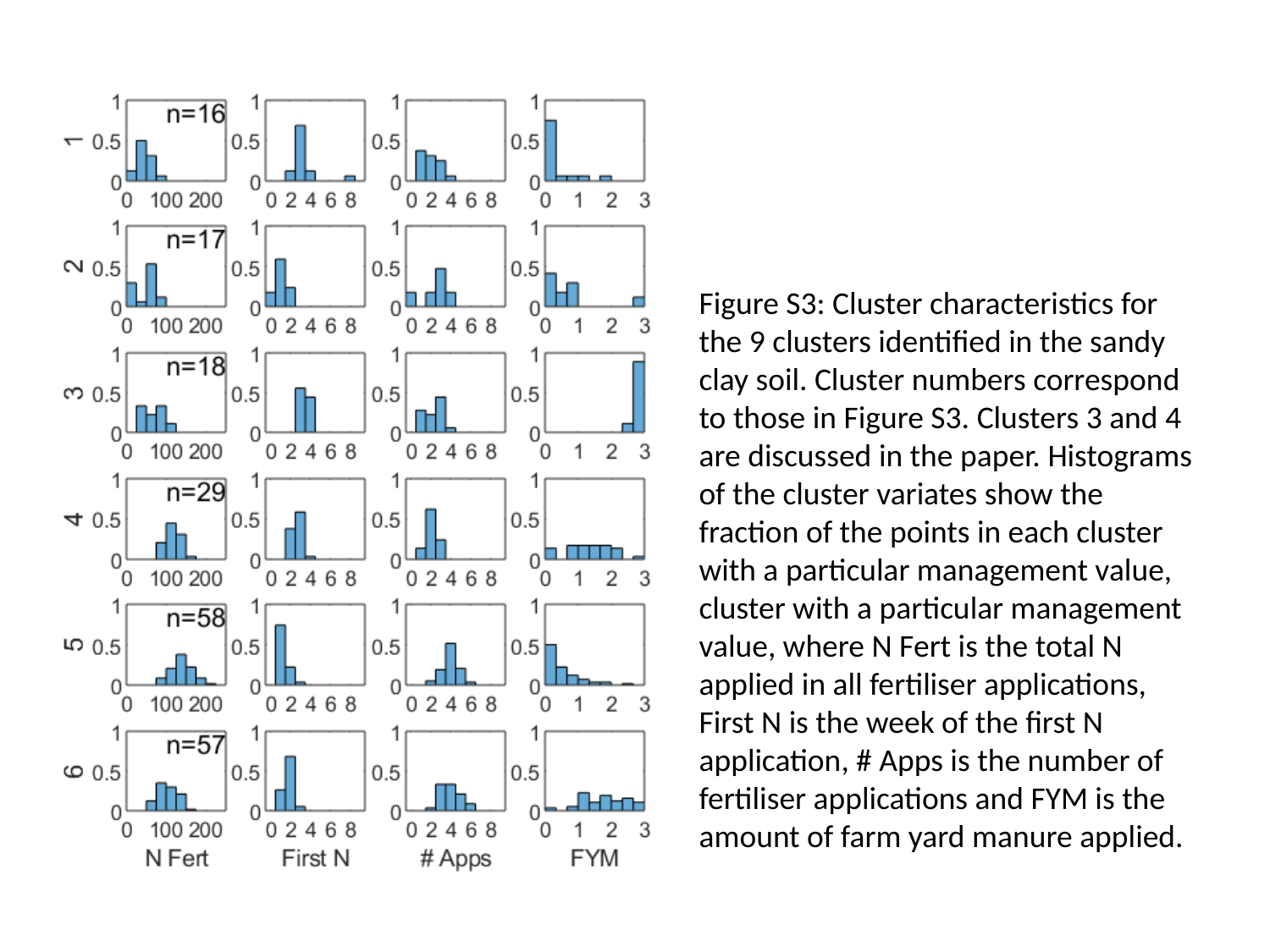

Figure S3: Cluster characteristics for the 9 clusters identified in the sandy clay soil. Cluster numbers correspond to those in Figure S3. Clusters 3 and 4 are discussed in the paper. Histograms of the cluster variates show the fraction of the points in each cluster with a particular management value, cluster with a particular management value, where N Fert is the total N applied in all fertiliser applications, First N is the week of the first N application, # Apps is the number of fertiliser applications and FYM is the amount of farm yard manure applied.

## Slide 5
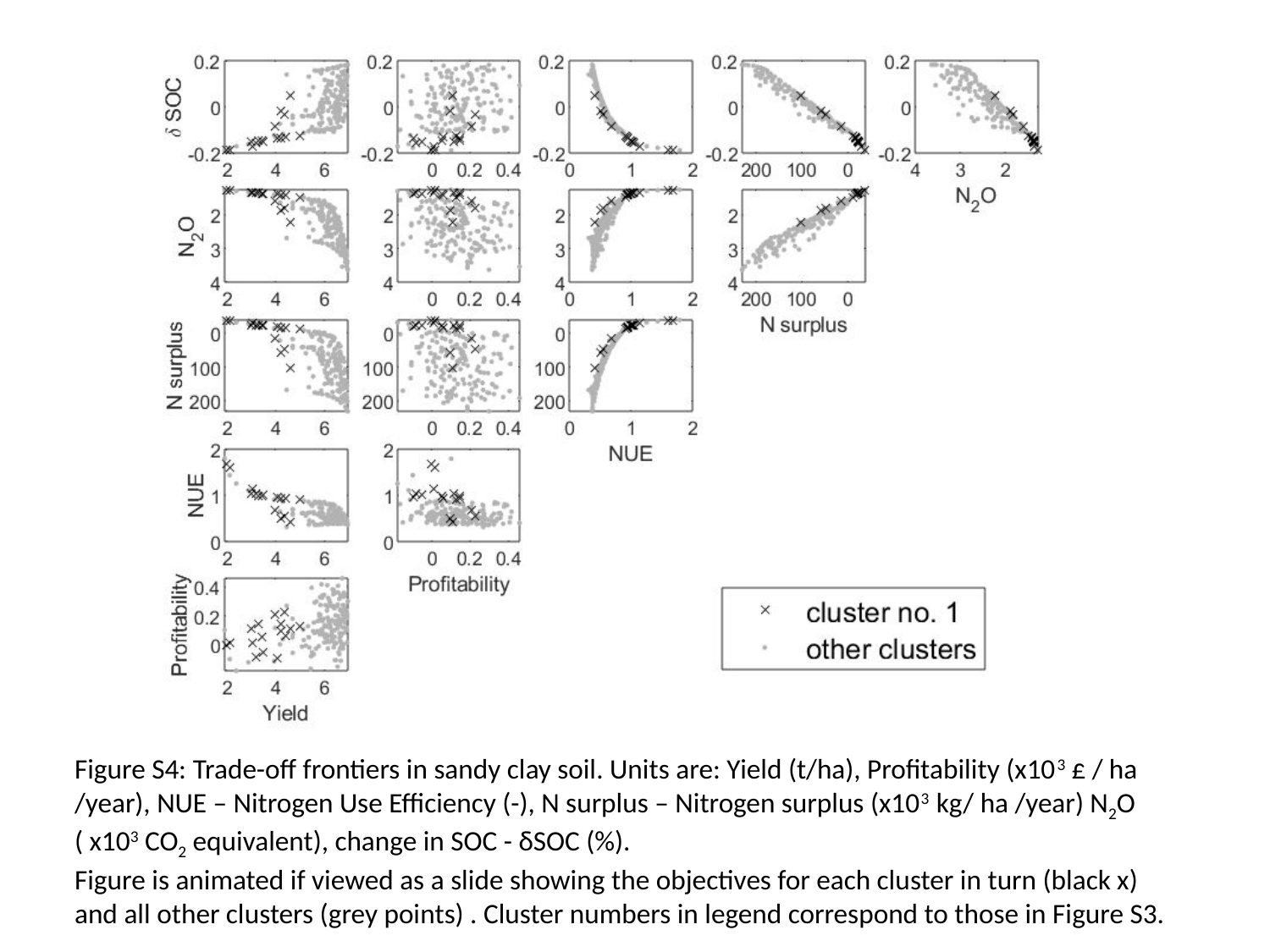

Figure S4: Trade-off frontiers in sandy clay soil. Units are: Yield (t/ha), Profitability (x103 £ / ha /year), NUE – Nitrogen Use Efficiency (-), N surplus – Nitrogen surplus (x103 kg/ ha /year) N2O ( x103 CO2 equivalent), change in SOC - δSOC (%).
Figure is animated if viewed as a slide showing the objectives for each cluster in turn (black x) and all other clusters (grey points) . Cluster numbers in legend correspond to those in Figure S3.

## Slide 6
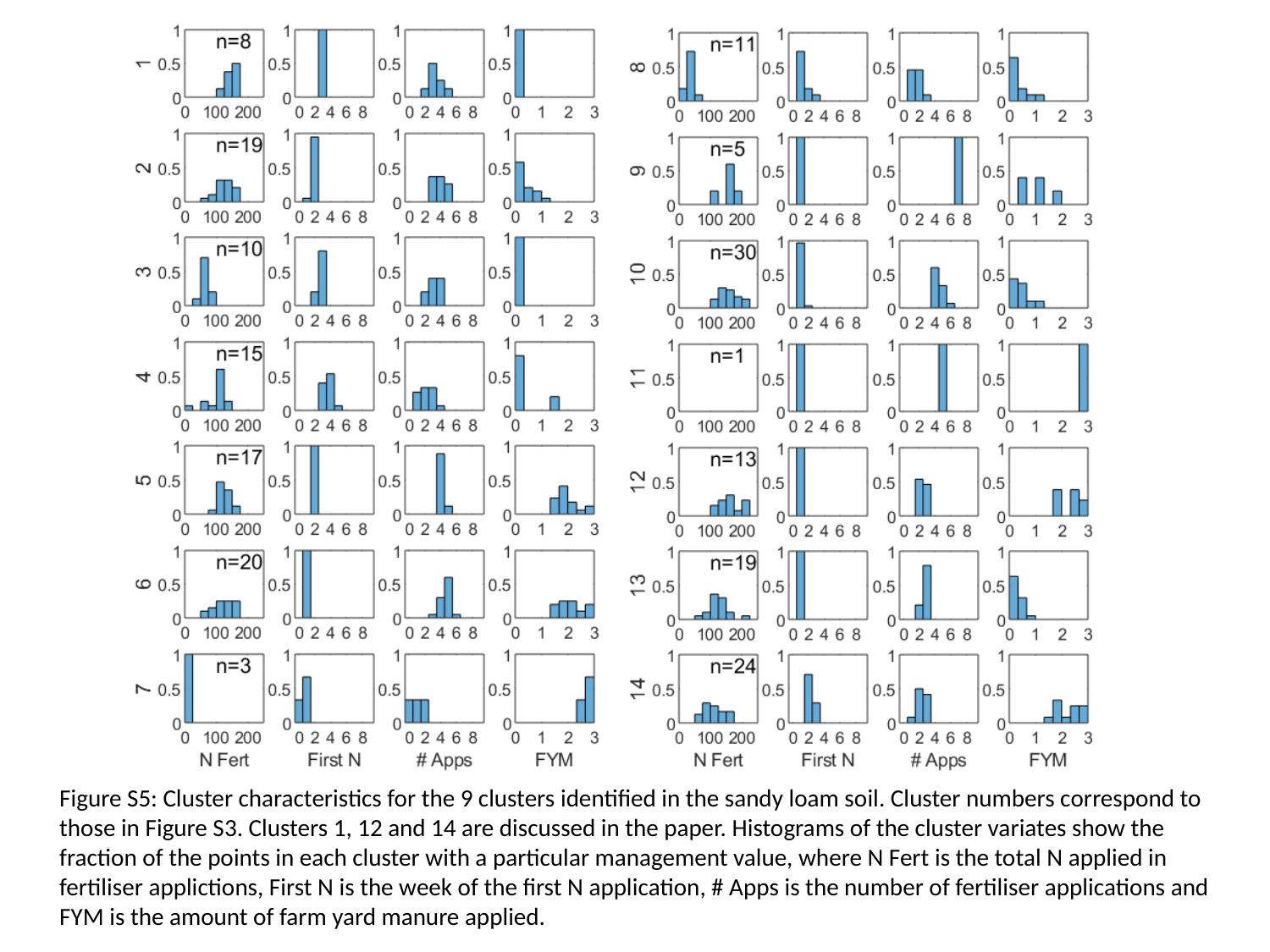

Figure S5: Cluster characteristics for the 9 clusters identified in the sandy loam soil. Cluster numbers correspond to those in Figure S3. Clusters 1, 12 and 14 are discussed in the paper. Histograms of the cluster variates show the fraction of the points in each cluster with a particular management value, where N Fert is the total N applied in fertiliser applictions, First N is the week of the first N application, # Apps is the number of fertiliser applications and FYM is the amount of farm yard manure applied.

## Slide 7
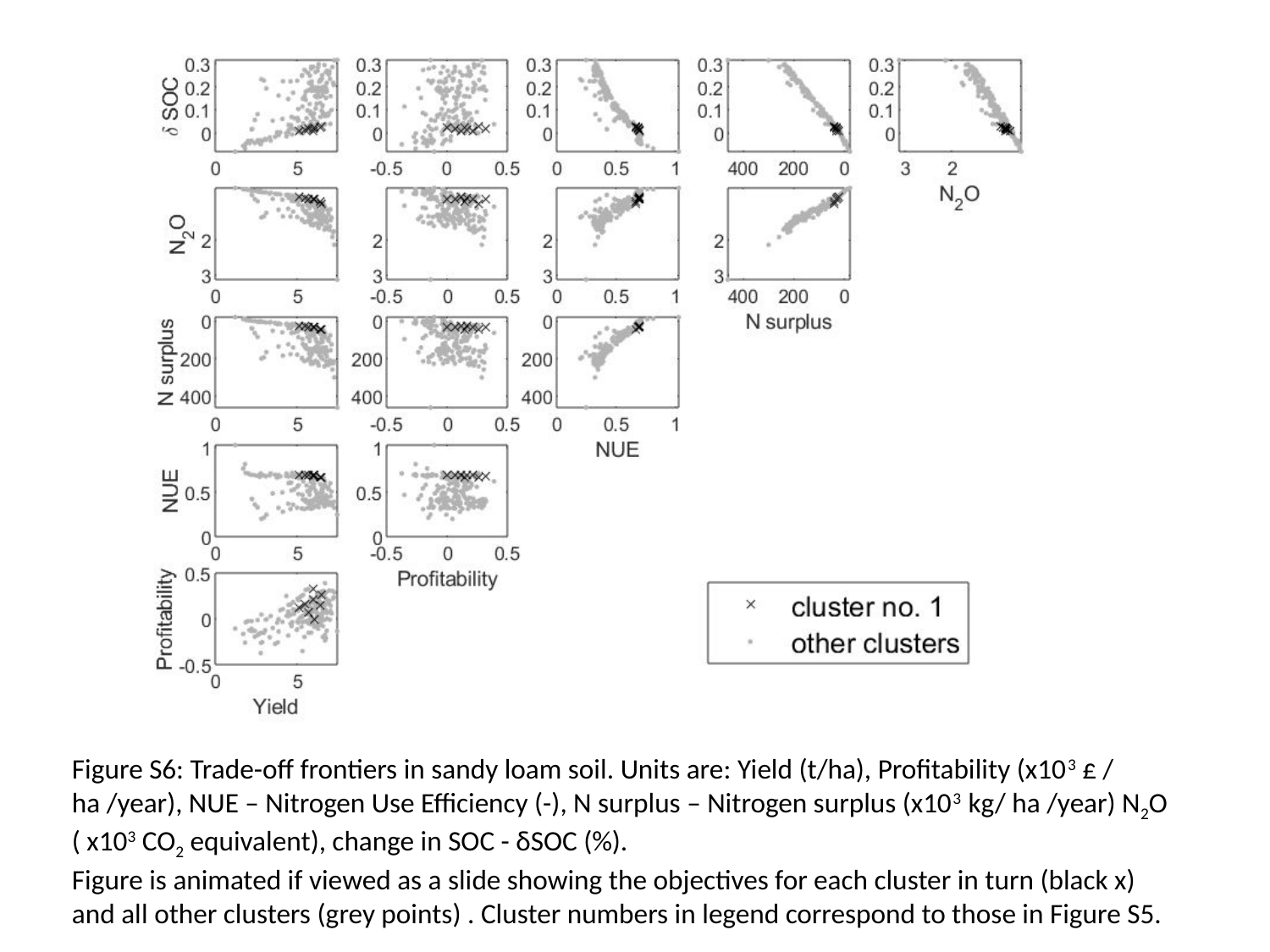

Figure S6: Trade-off frontiers in sandy loam soil. Units are: Yield (t/ha), Profitability (x103 £ / ha /year), NUE – Nitrogen Use Efficiency (-), N surplus – Nitrogen surplus (x103 kg/ ha /year) N2O ( x103 CO2 equivalent), change in SOC - δSOC (%).
Figure is animated if viewed as a slide showing the objectives for each cluster in turn (black x) and all other clusters (grey points) . Cluster numbers in legend correspond to those in Figure S5.
